# Supplementary material for: Gene expression profiling reveals insights into infant immunological and febrile responses to group B meningococcal vaccine
Source: Mol Syst Biol. 2020 Nov 19;16(11):e9888. doi: 10.15252/msb.20209888 (PMC7674973; doi:10.15252/msb.20209888)
Supplement: Supplementary file 1 — Appendix [file MSB-16-e9888-s001.pdf]

Supplementary Appendix

Table of Contents

APPENDIX 1
APPENDIX FIGURE S1: CONSORT DIAGRAM OF THE STUDY... 3
APPENDIX FIGURE S2: A) NEUTROPHIL COUNTS FOLLOWING INFANT VACCINATION IN FULL BLOOD COUNT. PLOTTED ARE THE NEUTROPHIL COUNTS, WITH MEDIAN AND INTERQUARTILE RANGE. P-VALUES WERE DETERMINED FROM A TWO SAMPLE WILCOXON RANK SUM TEST. B) SPEARMAN'S RANK CORRELATION BETWEEN NEUTROPHIL COUNTS MEASURE BY FULL BLOOD COUNTS AND THOSE ESTIMATED BY CIBERSORTX, USING THE IMMUNOSTATES BASIS MATRIX. 4
APPENDIX FIGURE S3: PRINCIPAL COMPONENT ANALYSIS DECONVOLUTED CELL FRACTIONS (N= 253, CIBERSORT, LM22) AND THE TOP CELL PHENOTYPES CONTRIBUTING TO PRINCIPAL COMPONENTS 1 AND 2. NOTE: CONTRIBUTION IS A PERCENTAGE, I.E. 50 IS 50% CONTRIBUTION THAT VARIABLE IS MAKING TO THE PRINCIPAL COMPONENTS 5
APPENDIX FIGURE S4: VOLCANO PLOT HIGHLIGHTING DIFFERENTIALLY EXPRESSED GENES (DEGS, FALSE-DISCOVERY RATE [FDR] <0.01), CORRECTED FOR CIBERSORT NEUTROPHIL FRACTION. A) 4 HOURS POST-VACCINATION (161 DEGS, N=28), B) 24 HOURS POST-VACCINATION (3228 DEGS, N=31), C) 3 DAYS POST-VACCINATION (125 DEGS, N=30), D) 7 DAYS POST-VACCINATION (6 DEGS, N=36). RED UPREGULATED AND BLUE DOWNREGULATED, THE TOP 10 GENES, RANKED BY FDR, ARE LABELLED. P-VALUES WERE OBTAINED FROM THE MODERATED T-STATISTIC, AFTER ADJUSTMENT FOR MULTIPLE TESTING (BENJAMINI AND HOCHBERG'S METHOD). 6
APPENDIX FIGURE S5: VENN DIAGRAMS OF OVERLAP IN DIFFERENTIAL EXPRESSED GENES (DEGS) IN 4CMENB PLUS CONTROL VACCINES (4CMENB+) AND CONTROL VACCINES ALONE (CTL). P-VALUES WERE OBTAINED FROM THE MODERATED T-STATISTIC, AFTER ADJUSTMENT FOR MULTIPLE TESTING (BENJAMINI AND HOCHBERG'S METHOD), DEGS (FALSE-DISCOVERY RATE [FDR] <0.01). A) CTL, N =18 AND 4CMENB+, N= 10; B) CTL, N =16 AND 4CMENB+, N= 15; C) CTL, N =16 AND 4CMENB+, N= 14; D) CTL, N =17 AND 4CMENB+, N= 19. 7
APPENDIX FIGURE S6: AGREEMENT PLOT. GREEN = DIFFERENTIALLY EXPRESSED (DE) FOLLOWING CONTROL VACCINES ONLY, PURPLE = DE AFTER 4CMENB + CONTROL VACCINES ONLY, CYAN = DE IN BOTH VACCINE GROUPS. P-VALUES WERE OBTAINED FROM THE MODERATED T-STATISTIC, AFTER ADJUSTMENT FOR MULTIPLE TESTING (BENJAMINI AND HOCHBERG'S METHOD), DEGS (FALSE-DISCOVERY RATE [FDR] <0.01). A) CTL, N =18 AND 4CMENB+, N= 10; B) CTL, N =16 AND 4CMENB+, N= 15; C) CTL, N =16 AND 4CMENB+, N= 14; D) CTL, N =17 AND 4CMENB+, N= 19. 8
APPENDIX FIGURE S7: SIGNIFICANTLY ENRICHED GENE ONTOLOGIES ASSOCIATED WITH PARTICULAR BIOLOGICAL PROCESS. GENE SET ENRICHMENT ANALYSIS WAS PERFORMED ON DIFFERENTIALLY EXPRESSED GENES (FDR <0.01), AFTER ADJUSTMENT OF P-VALUES WERE OBTAINED FROM THE MODERATED T-STATISTIC USING THE BENJAMINI AND HOCHBERG'S METHOD. ENRICHMENT ANALYSIS WAS BASED ON A HYPERGEOMETRIC TEST. 4HR CONTROL, N= 18; 4HR 4CMENB+, N= 10; 24HR CONTROL, N =16 AND 24HR 4CMENB+, N= 15. 9
APPENDIX FIGURE S8: SIGNIFICANTLY ENRICHED GENE ONTOLOGIES ASSOCIATED WITH PARTICULAR MOLECULAR FUNCTIONS. GENE SET ENRICHMENT ANALYSIS WAS PERFORMED ON DIFFERENTIALLY EXPRESSED GENES (FDR <0.01), AFTER ADJUSTMENT OF P-VALUES WERE OBTAINED FROM THE MODERATED T-STATISTIC USING THE BENJAMINI AND HOCHBERG'S METHOD. ENRICHMENT ANALYSIS WAS BASED ON A HYPERGEOMETRIC TEST. 4HR CONTROL, N= 18; 4HR 4CMENB+, N= 10; 24HR CONTROL, N =16 AND 24HR 4CMENB+, N= 15. 10
APPENDIX FIGURE S9: SIGNIFICANTLY ENRICHED GENE ONTOLOGIES ASSOCIATED WITH PARTICULAR CELLULAR COMPONENTS. GENE SET ENRICHMENT ANALYSIS WAS PERFORMED ON DIFFERENTIALLY EXPRESSED GENES

Formatted: Position: Horizontal: Right, Relative to: Margin, Vertical: 0", Relative to: Paragraph, Wrap Around

(FDR <0.01), after adjustment of p-values were obtained from the moderated t-statistic using the Benjamini and Hochberg's method. Enrichment analysis was based on a hypergeometric test. 4hr control, n= 18; 4hr 4CMenB+, n= 10; 24hr control, n =16 and 24hr 4CMenB+, n= 15. 11

Appendix Figure S10: Matrix showing Spearman's rank correlations between plasma cytokine levels and cellular proportions (determined by CIBERSORT), only correlations with an associated p-value <0.01 are displayed (n=58 for IL1RA, IL5, IL6 and GCSF; n=61 for CRP and n= 253 for cell frequencies). Correlation analysis was limited to plasma cytokines that significantly changed post-vaccination (FDR <0.05) and cellular population with median CIBERSORT proportions >5%. 12

Appendix Figure S11: CIBERSORT neutrophil fraction in whole blood samples following vaccination. Infants who had a temperature measurement above 38 are classified as having had a fever. Plotted are the median and interquartile range. b) Post-vaccination MenB-specific SBA titres in infants who did, or did not, experience a fever event within 24 hours of vaccination (4CMenB+ group only). P-values were determined from a two sample Wilcoxon rank sum test. 13

Appendix Figure S12: a) Variance explained by the first two principal components of the baseline blood transcriptomics data (4CMenB vaccine group only, n=54), b) Performance of training model built with sparse distance weighted discrimination (sdwd) algorithm to predict development of fever after MenB vaccination using baseline blood gene expression, in the test cohort of infants (n=10)... 13

Appendix Figure S13: a) Overlap between significantly differentially expressed genes in the 4CMenB and control immunisation groups. Upset plot depicting the proportion of significantly differentially expressed genes (DEGs, FDR < 0.01) unique or common to each of the 3 experimental groups, obtained from the moderated t-statistic using the Benjamini and Hochberg's method. The total number of significantly DEGs are represented by the horizontal bars. The number of uniquely significantly DEGs are represented by the vertical bars above the individual dots corresponding to each group, while the number of commonly significantly DEGs are represented above the dots joined by a vertical line beneath. b) Comparison of significantly enriched immunological pathways (Reactome database) 4CMenB + control immunisation or 4CMenB alone. c) Enriched pathway analysis using cell signalling pathway categories from the publicly available integrating network objects with hierarchies (INOH) database. The dashed red line corresponds to a negative log_{10}pathway adjusted (FDR) p-value of 0.05. Enrichment analysis was based on a hypergeometric test. n =6 mice per group........... 16

Appendix Figure S14: Heatmap of cell deconvolution analysis performed on whole blood gene signatures 24 hours after immunisation with 4CMenB and control immunisations. Inferred relative quantity (IRQ) for each immune cell population (y-axis) are represented as log2 fold-change values corresponding to each tile of the heatmaps. Heatmaps are faceted by immune cell category. n = 6 mice per group. 17

Appendix Figure S15: Enrichment of the neutrophils from blood subset of granulocyte fraction from the cell deconvolution analysis. Relative abundance of neutrophils in whole blood based inferred from log_2 fold-changes of genes identified for this phenotype from the Immunological Genome Project (ImmGen) database. Significant differences between groups were determined using a two sample Wilcoxon rank sum test. n=6 mice per group. Vertical lines represent the 95% confidence interval around the mean. 18

Formatted: Position: Horizontal: Right, Relative to: Margin, Vertical: 0", Relative to: Paragraph, Wrap Around

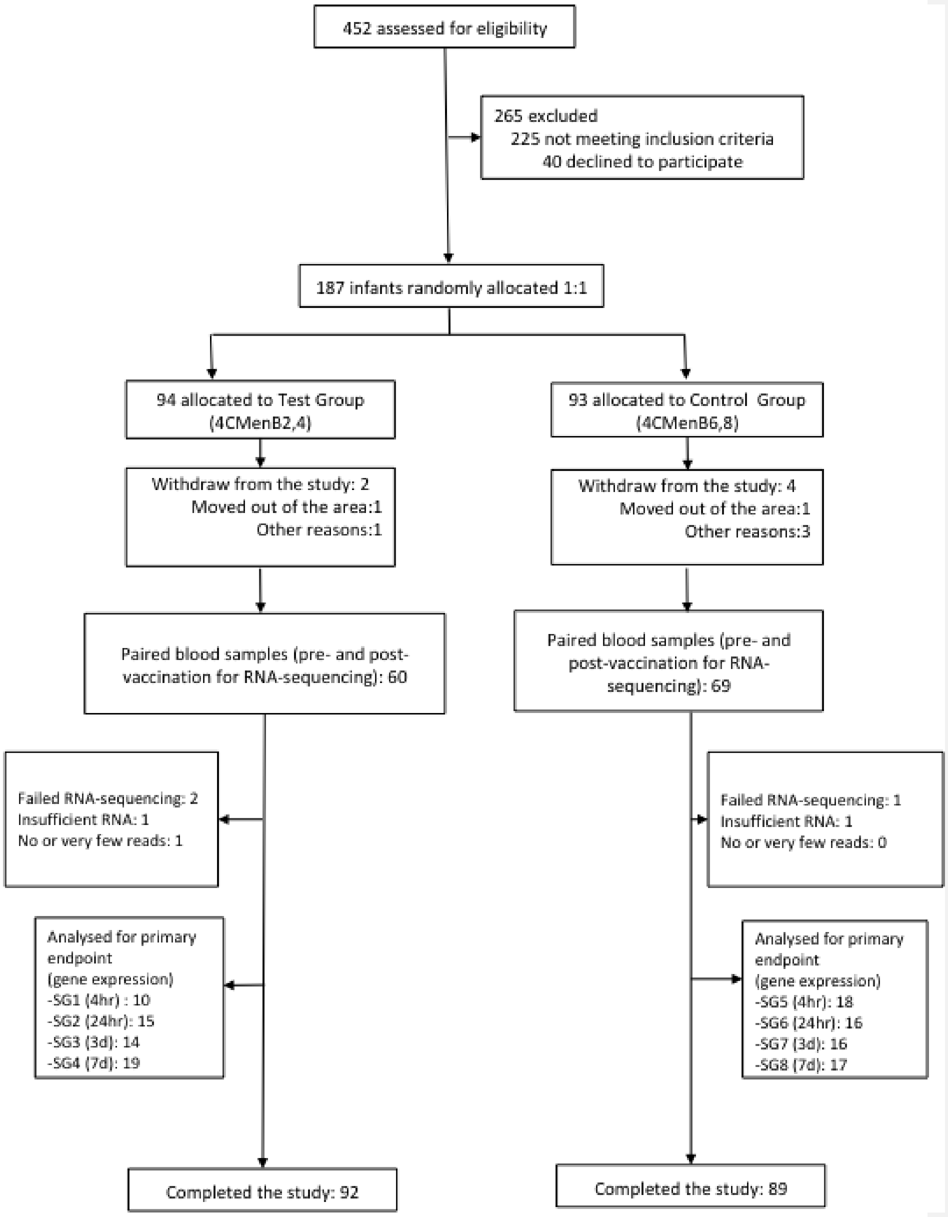

Appendix Figure S1: Consort diagram of the study.

Formatted: Position: Horizontal: Right, Relative to: Margin, Vertical: 0", Relative to: Paragraph, Wrap Around

a) b)

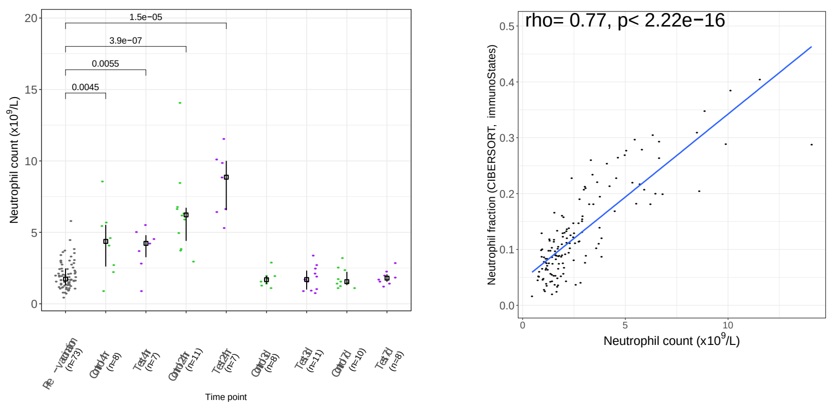

Appendix Figure S2: a) Neutrophil counts following infant vaccination in full blood count. Plotted are the neutrophil counts, with median and interquartile range. P-values were determined from a two sample Wilcoxon rank sum test. b) Spearman's rank correlation between neutrophil counts measure by full blood counts and those estimated by CIBERSORTx, using the immunoStates basis matrix.

Formatted: Position: Horizontal: Right, Relative to: Margin, Vertical: 0", Relative to: Paragraph, Wrap Around

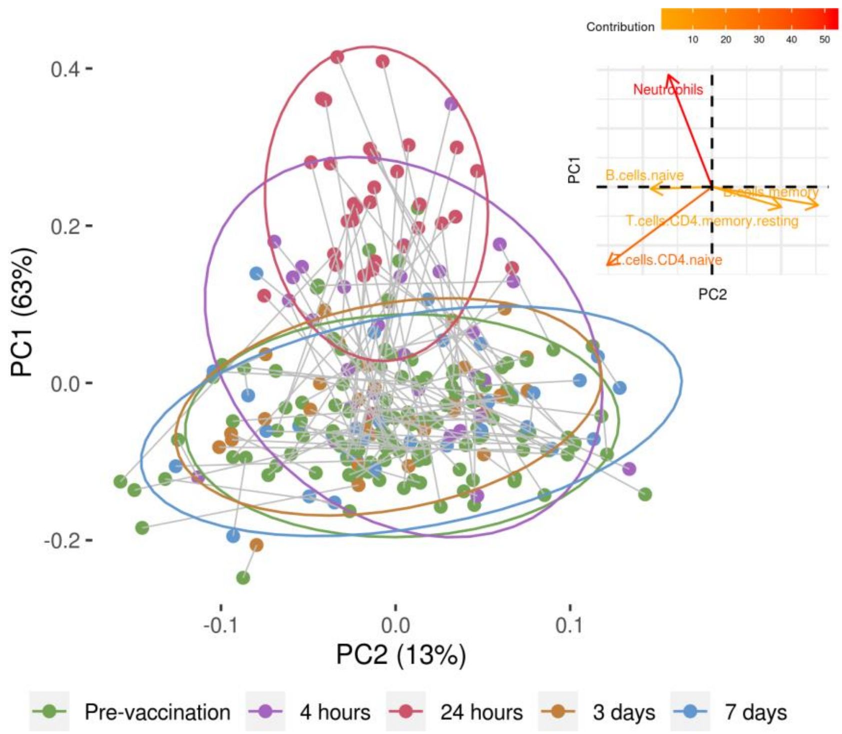

Appendix Figure S3: Principal component analysis deconvoluted cell fractions (n= 253, CIBERSORT, LM22) and the top cell phenotypes contributing to principal components 1 and 2. Note: contribution is a percentage, i.e. 50 is 50% contribution that variable is making to the principal components .

Formatted: Position: Horizontal: Right, Relative to: Margin, Vertical: 0", Relative to: Paragraph, Wrap Around

Supplementary figure 3: Principal component analysis deconvoluted cell fractions (CIBERSORT, LM22) and the top

Appendix Figure S4: Volcano plot highlighting differentially expressed genes (DEGs, false-discovery rate [FDR] <0.01), corrected for CIBERSORT neutrophil fraction. a) 4 hours post-vaccination (161 DEGs, n=28), b) 24 hours post-vaccination (3228 DEGs, n=31), c) 3 days post-vaccination (125 DEGs, n=30), d) 7 days post-vaccination (6 DEGs, n=36). Red upregulated and blue downregulated, the top 10 genes, ranked by FDR, are labelled. P-values were obtained from the moderated t-statistic, after adjustment for multiple testing (Benjamini and Hochberg's method).

Formatted: Position: Horizontal: Right, Relative to: Margin, Vertical: 0", Relative to: Paragraph, Wrap Around

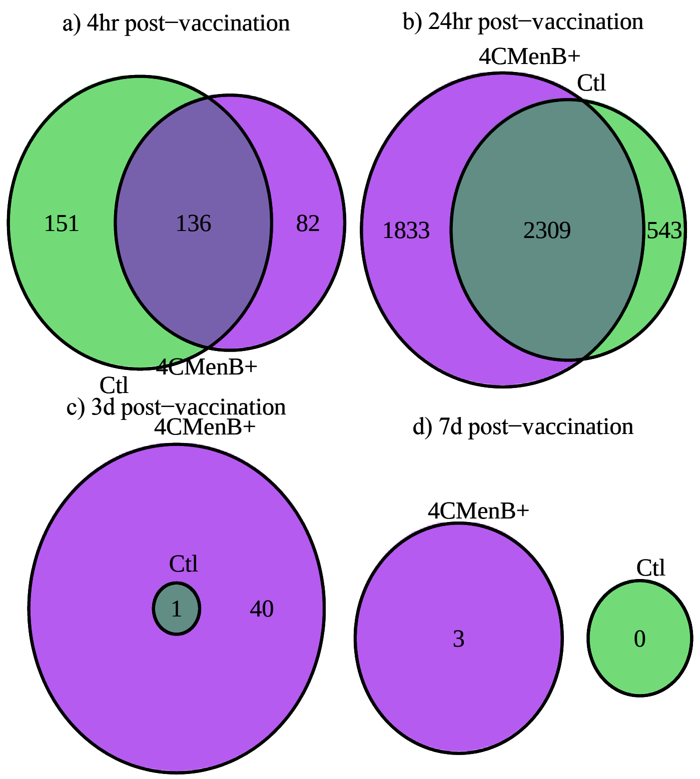

Appendix Figure S5: Venn diagrams of overlap in differential expressed genes (DEGs) in 4CMenB plus control vaccines (4CMenB+) and control vaccines alone (Ctl). P-values were obtained from the moderated t-statistic, after adjustment for multiple testing (Benjamini and Hochberg's method), DEGs (false-discovery rate [FDR] <0.01). a) Ctl, n =18 and 4CMenB+, n= 10; b) Ctl, n =16 and 4CMenB+, n= 15; c) Ctl, n =16 and 4CMenB+, n= 14; d) Ctl, n =17 and 4CMenB+, n= 19.

Formatted: Position: Horizontal: Right, Relative to: Margin, Vertical: 0", Relative to: Paragraph, Wrap Around

Supplementary figure 5: Venn diagrams of overlap in differential expressed genes in 4CMenB plus control vaccines

Appendix Figure S6: Agreement plot. Green = differentially expressed (DE) following control vaccines only, purple = DE after 4CMenB + control vaccines only, cyan = DE in both vaccine groups. P-values were obtained from the moderated t-statistic, after adjustment for multiple testing (Benjamini and Hochberg's method), DEGs (false-discovery rate [FDR] <0.01). a) Ctl, n =18 and 4CMenB+, n= 10; b) Ctl, n =16 and 4CMenB+, n= 15; c) Ctl, n =16 and 4CMenB+, n= 14; d) Ctl, n =17 and 4CMenB+, n= 19.

Supplementary figure 6: Agreement plot. Green = differentially expressed (DE) following control vaccines only, purple = DE after 4CMenB + control vaccines only, cyan = DE in both vaccine groups.

Formatted: Position: Horizontal: Right, Relative to: Margin, Vertical: 0", Relative to: Paragraph, Wrap Around

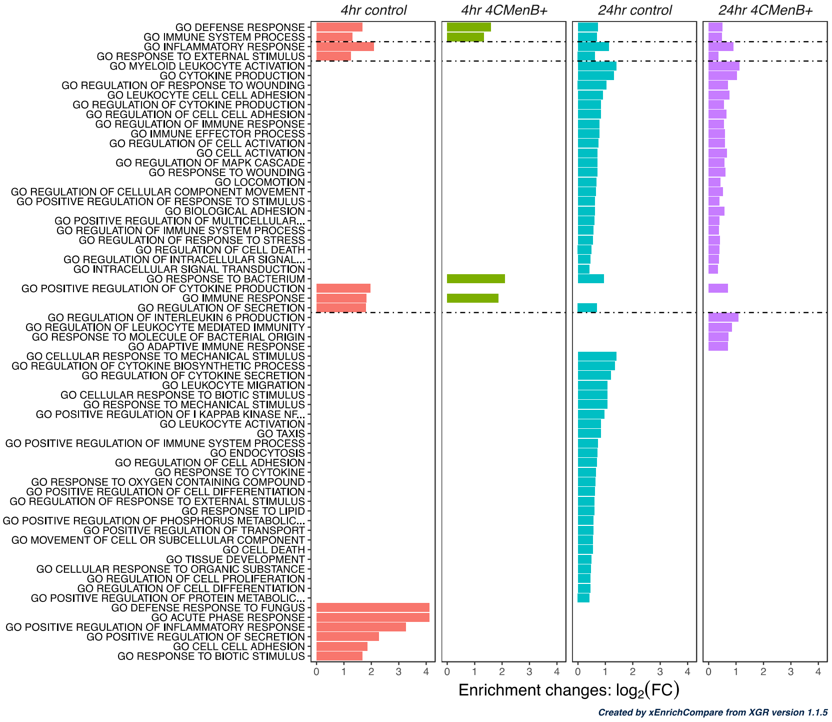

Appendix Figure S7: Significantly enriched gene ontologies associated with particular biological process. Gene set enrichment analysis was performed on differentially expressed genes (FDR <0.01), after adjustment of p-values were obtained from the moderated t-statistic using the Benjamini and Hochberg's method. Enrichment analysis was based on a hypergeometric test. 4hr control, n= 18; 4hr 4CMenB+, n= 10; 24hr control, n =16 and 24hr 4CMenB+, n= 15.

~~**Supplementary figure 7: Significantly enriched gene ontologies associated with particular biological process.**~~

Formatted: Position: Horizontal: Right, Relative to: Margin, Vertical: 0", Relative to: Paragraph, Wrap Around

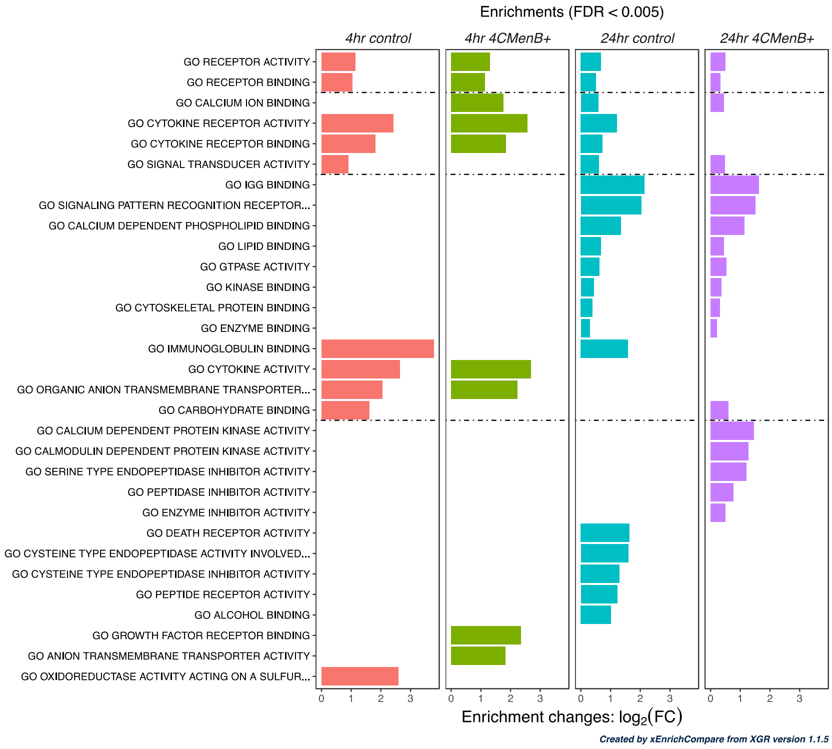

Appendix Figure S8: Significantly enriched gene ontologies associated with particular molecular functions. Gene set enrichment analysis was performed on differentially expressed genes (FDR <0.01), after adjustment of p-values were obtained from the moderated t-statistic using the Benjamini and Hochberg's method. Enrichment analysis was based on a hypergeometric test. 4hr control, n= 18; 4hr 4CMenB+, n= 10; 24hr control, n =16 and 24hr 4CMenB+, n= 15.

Formatted: Position: Horizontal: Right, Relative to: Margin, Vertical: 0", Relative to: Paragraph, Wrap Around

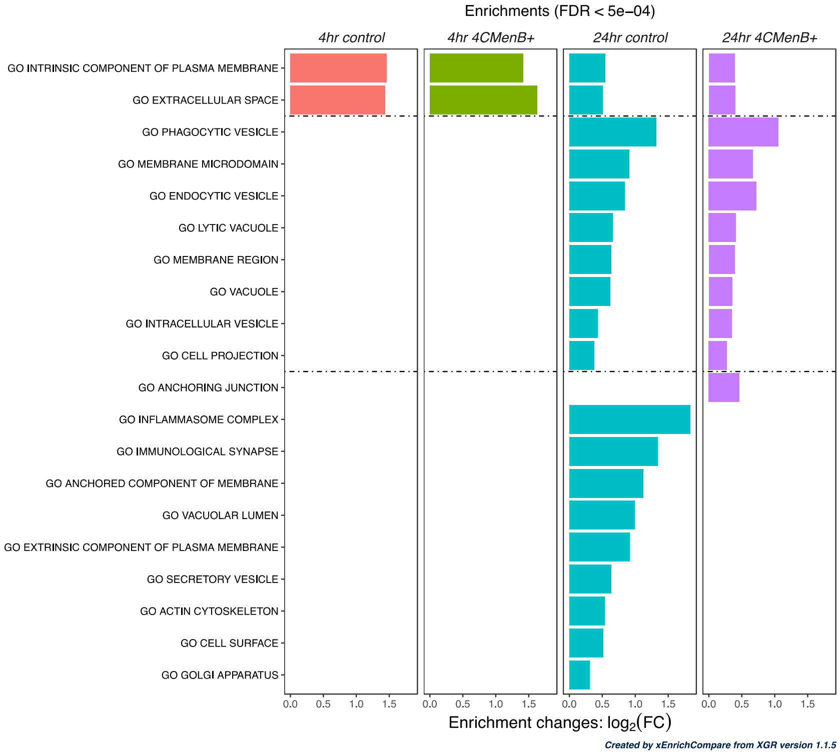

Appendix Figure S9: Significantly enriched gene ontologies associated with particular cellular components. Gene set enrichment analysis was performed on differentially expressed genes (FDR <0.01), after adjustment of p-values were obtained from the moderated t-statistic using the Benjamini and Hochberg's method. Enrichment analysis was based on a hypergeometric test. 4hr control, n= 18; 4hr 4CMenB+, n= 10; 24hr control, n =16 and 24hr 4CMenB+, n= 15.

~~**Supplementary figure 9: Significantly enriched gene ontologies associated with particular cellular components.**~~

Formatted: Heading 3, Don't keep with next

Formatted: Font: (Default) Times New Roman, 9 pt, Font color: Accent 1

Formatted: Font: (Default) Times New Roman, 9 pt, Font color: Accent 1

Formatted: Font: (Default) Times New Roman, 9 pt, Font color: Accent 1

Formatted: Position: Horizontal: Right, Relative to: Margin, Vertical: 0", Relative to: Paragraph, Wrap Around

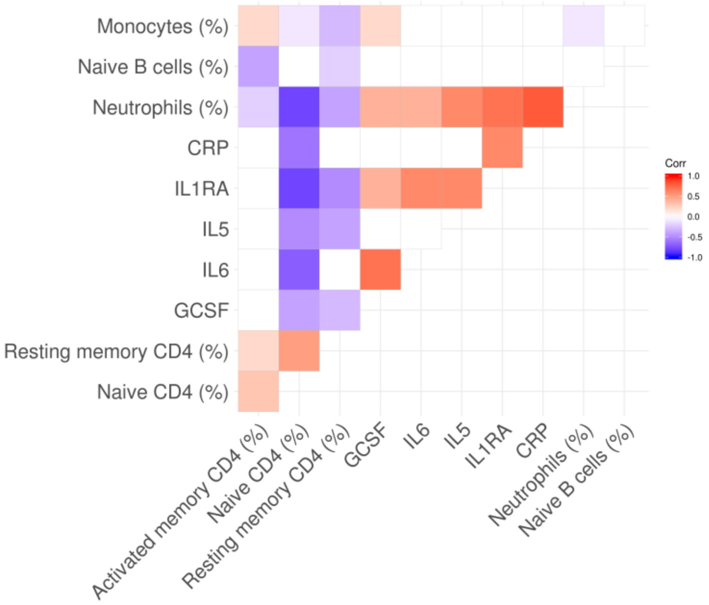

Appendix Figure S10: Matrix showing Spearman's rank correlations between plasma cytokine levels and cellular proportions (determined by CIBERSORT), only correlations with an associated p-value <0.01 are displayed (n=58 for IL1RA, IL5, IL6 and GCSF; n=61 for CRP and n= 253 for cell frequencies). Correlation analysis was limited to plasma cytokines that significantly changed post-vaccination (FDR <0.05) and cellular population with median CIBERSORT proportions >5%.

Supplementary figure 10: Matrix showing Spearman's rank correlations between plasma cytokine levels and cellular proportions (determined by CIBERSORT), only correlations with an associated p-value <0.01 are displayed. Correlation analysis was limited to plasma cytokines that significantly changed post-vaccination (FDR <0.05) and cellular population with median CIBERSORT proportions >5%.

Formatted: Position: Horizontal: Right, Relative to: Margin, Vertical: 0", Relative to: Paragraph, Wrap Around

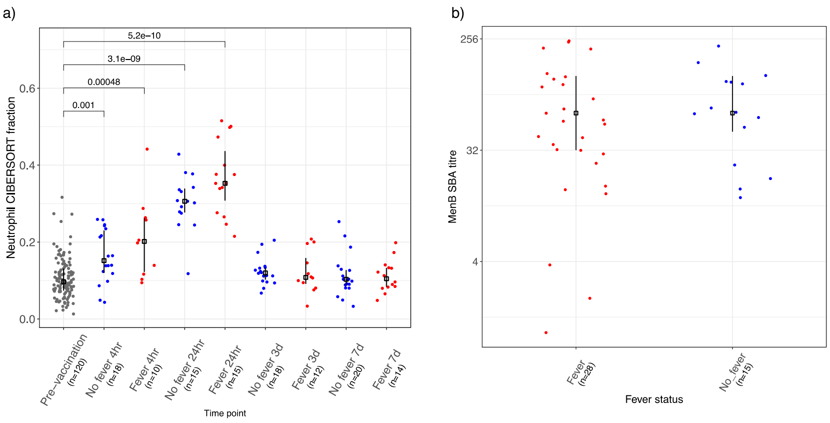

Appendix Figure S11: CIBERSORT neutrophil fraction in whole blood samples following vaccination. Infants who had a temperature measurement above 38 are classified as having had a fever. Plotted are the median and interquartile range. b) Post-vaccination MenB-specific SBA titres in infants who did, or did not, experience a fever event within 24 hours of vaccination (4CMenB+ group only). P-values were determined from a two sample Wilcoxon rank sum test.

~~**Supplementary figure 11: CIBERSORT neutrophil fraction in whole blood samples following vaccination. Infants who had a temperature measurement above 38 are classified as having had a fever. Plotted are the median and interquartile range. b) Post-vaccination MenB-specific SBA titres in infants who did, or did not, experience a fever event within 24 hours of vaccination (4CMenB+ group only). P-values were determined from a two sample Wilcoxon rank sum test.**~~

Appendix Figure S12: a) Variance explained by the first two principal components of the baseline blood transcriptomics data (4CMenB vaccine group only, n=54), b) Performance of training model built with sparse distance weighted discrimination (sdwd) algorithm to predict development of fever after MenB vaccination using baseline blood gene expression, in the test cohort of infants (n=10) (as depicted in Figure 6d).

~~**Supplementary figure 12: a) Variance explained by the first two principal components of the baseline blood transcriptomics data (4CMenB vaccine group only), b) Performance of training model built with sparse distance weighted discrimination (sdwd) algorithm to predict development of fever after MenB vaccination using baseline blood gene expression, in the test cohort of infants (n=10).**~~

Formatted: Position: Horizontal: Right, Relative to: Margin, Vertical: 0", Relative to: Paragraph, Wrap Around

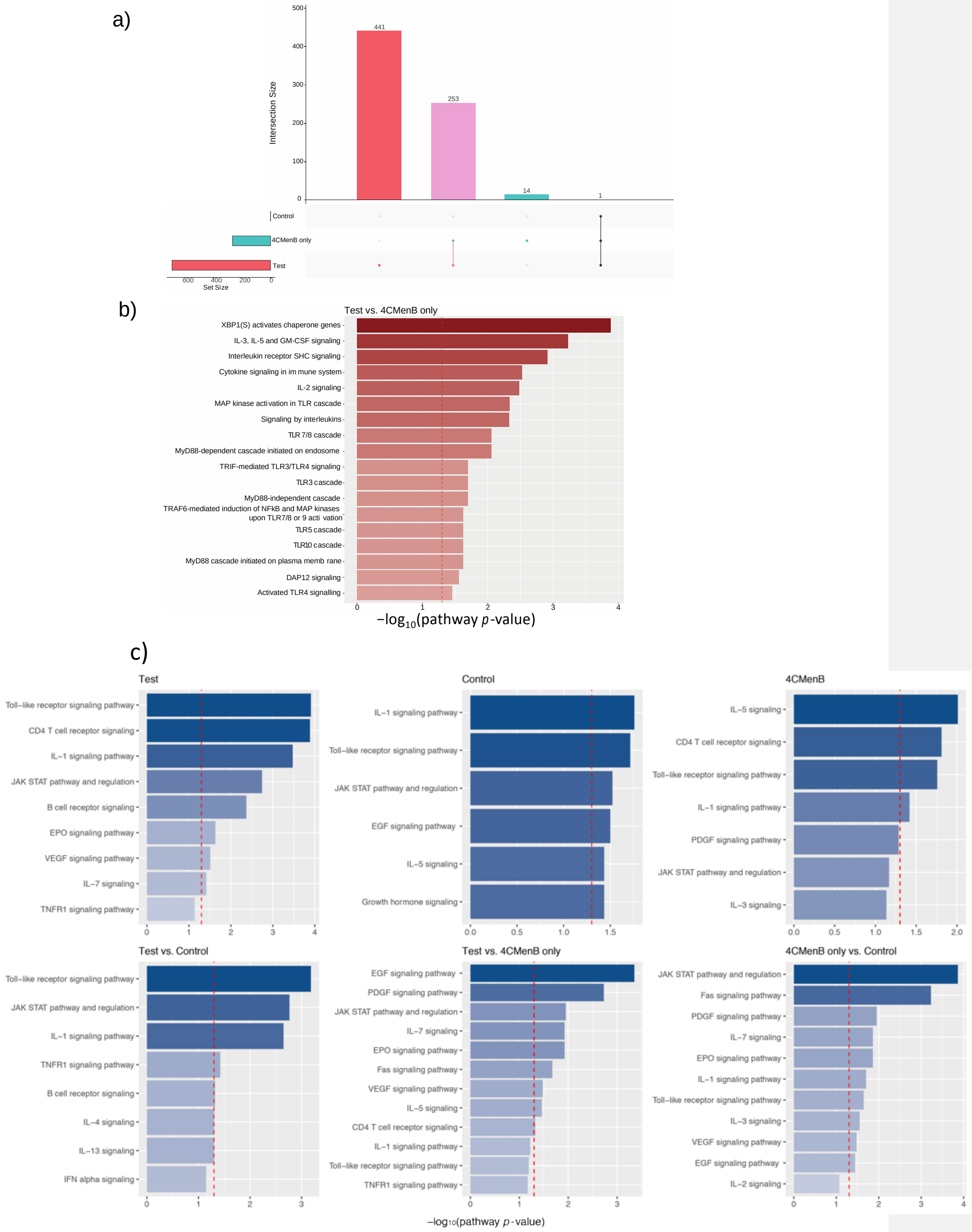

Formatted: Position: Horizontal: Right, Relative to: Margin, Vertical: 0", Relative to: Paragraph, Wrap Around

Appendix Figure S13: a) Overlap between significantly differentially expressed genes in the 4CMenB and control immunisation groups. Upset plot depicting the proportion of significantly differentially expressed genes (DEGs, FDR < 0.01) unique or common to each of the 3 experimental groups, obtained from the moderated t-statistic using the Benjamini and Hochberg's method. The total number of significantly DEGs are represented by the horizontal bars. The number of uniquely significantly DEGs are represented by the vertical bars above the individual dots corresponding to each group, while the number of commonly significantly DEGs are represented above the dots joined by a vertical line beneath. b) Comparison of significantly enriched immunological pathways (Reactome database) 4CMenB + control immunisation or 4CMenB alone. c) Enriched pathway analysis using cell signalling pathway categories from the publicly available integrating network objects with hierarchies (INOH) database. The dashed red line corresponds to a negative log_{10}pathway adjusted (FDR) p-value of 0.05. Enrichment analysis was based on a hypergeometric test. n =6 mice per group.

Supplementary figure 13: a) Overlap between significantly differentially expressed genes in the 4CMenB and control immunisation groups. Upset plot depicting the proportion of significantly differentially expressed genes (DEGs, FDR < 0.01) unique or common to each of the 3 experimental groups. The total number of significantly DEGs are represented by the horizontal bars. The number of uniquely significantly DEGs are represented by the vertical bars above the individual dots corresponding to each group, while the number of commonly significantly DEGs are represented above the dots joined by a vertical line beneath. b) Comparison of significantly enriched immunological pathways (Reactome database) 4CMenB + control immunisation or 4CMenB alone. c) Enriched pathway analysis using cell signalling pathway categories from the publicly available integrating network objects with hierarchies (INOH) database. The dashed red line corresponds to a negative log_{10}pathway adjusted (FDR) p-value of 0.05.

Formatted: Position: Horizontal: Right, Relative to: Margin, Vertical: 0", Relative to: Paragraph, Wrap Around

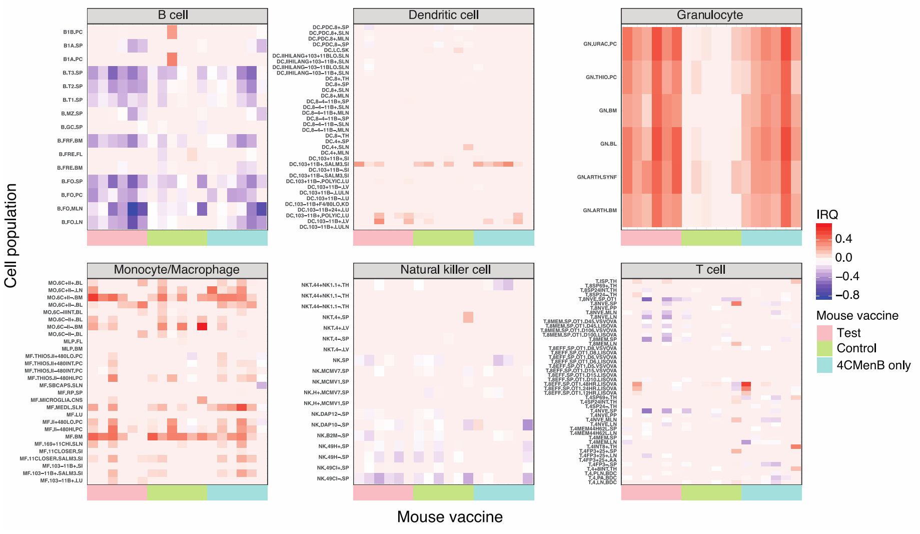

Appendix Figure S14: Heatmap of cell deconvolution analysis performed on whole blood gene signatures 24 hours after immunisation with 4CMenB and control immunisations. Inferred relative quantity (IRQ) for each immune cell population (y-axis) are represented as log2 fold-change values corresponding to each tile of the heatmaps. Heatmaps are faceted by immune cell category. n = 6 mice per group.

~~**Supplementary figure 14: Heatmap of cell deconvolution analysis performed on whole blood gene signatures 24 hours after immunisation with 4CMenB and control immunisations. Inferred relative quantity (IRQ) for each immune cell**~~

Formatted: Position: Horizontal: Right, Relative to: Margin, Vertical: 0", Relative to: Paragraph, Wrap Around

~~population (y-axis) are represented as log2 fold-change values corresponding to each tile of the heatmaps. Heatmaps~~

Appendix Figure S15: Enrichment of the neutrophils from blood subset of granulocyte fraction from the cell deconvolution analysis. Relative abundance of neutrophils in whole blood based inferred from log_2 fold-changes of genes identified for this phenotype from the Immunological Genome Project (ImmGen) database. Significant differences between groups were determined using a two sample Wilcoxon rank sum test. n=6 mice per group. Vertical lines represent the 95% confidence interval around the mean.

~~**Supplementary figure 15: Enrichment of the neutrophils from blood subset of granulocyte fraction from the cell deconvolution analysis. Relative abundance of neutrophils in whole blood based inferred from log_2 fold-changes of genes identified for this phenotype from the Immunological Genome Project (ImmGen) database. Significant differences between groups were determined using a two sample Wilcoxon rank sum test.**~~

Formatted: Position: Horizontal: Right, Relative to: Margin, Vertical: 0", Relative to: Paragraph, Wrap Around
